# Heterogeneous gene duplications can be adaptive because they permanently associate overdominant alleles

**DOI:** 10.1002/evl3.17

**Published:** 2017-07-21

**Authors:** Pascal Milesi, Mylène Weill, Thomas Lenormand, Pierrick Labbé

**Affiliations:** ^1^ Institut des Sciences de l'Evolution de Montpellier (UMR 5554, CNRS‐Université de Montpellier‐IRD‐EPHE) Campus Université de Montpellier Place Eugène Bataillon 34095 Montpellier CEDEX 05 France; ^2^ Centre d'Ecologie Fonctionnelle et Evolutive (UMR 5175, CNRS‐Université de Montpellier‐Université Paul‐Valéry Montpellier‐EPHE) 1919 route de Mende F‐34293 Montpellier CEDEX 05 France

**Keywords:** genetic polymorphism, gene duplication, genome evolution, overdominance, balancing selection, insecticide resistance

## Abstract

Gene duplications are widespread in genomes, but their role in contemporary adaptation is not fully understood. Although mostly deleterious, homogeneous duplications that associate identical repeats of a locus often increase the quantity of protein produced, which can be selected in certain environments. However, another type exists: heterogeneous gene duplications, which permanently associate two (or more) alleles of a single locus on the same chromosome. They are far less studied, as only few examples of contemporary heterogeneous duplications are known. Haldane proposed in 1954 that they could be adaptive in situations of heterozygote advantage, or overdominance, but this hypothesis was never tested. To assess its validity, we took advantage of the well‐known model of insecticide resistance in mosquitoes. We used experimental evolution to estimate the fitnesses associated with homozygous and heterozygous genotypes in different selection regimes. It first showed that balanced antagonist selective pressures frequently induce overdominance, generating stable polymorphic equilibriums. The frequency of equilibrium moreover depends on the magnitude of two antagonistic selective pressures, the survival advantage conferred by the resistant allele versus the selective costs it induces. We then showed that heterogeneous duplications are selected over single‐copy alleles in such contexts. They allow the fixation of the heterozygote phenotype, providing an alternative and stable intermediate fitness trade‐off. By allowing the rapid fixation of divergent alleles, this immediate advantage could contribute to the rarity of overdominance. More importantly, it also creates new material for long‐term genetic innovation, making a crucial but underestimated contribution to the evolution of new genes and gene families.

**Impact Summary**

Understanding the maintenance of polymorphism in natural populations despite the erosion due to natural selection and genetic drift has been one of the challenges of the early 20th century. One of the propositions, rapidly put aside due to the lack of examples was overdominance: when the heterozygote phenotype is the fittest, none of the alleles can fix and only half of the progeny of two heterozygotes carry the best phenotype (this is called segregation burden). However, in 1954 Haldane suggested that heterogeneous duplications associating the two alleles on the same chromosome could be selected, but could also allow the fixation of the heterozygote phenotype. This hypothesis was however never tested, because both overdominance and gene duplications were considered too rare. Recent genome‐wide studies showed that duplications are actually more common than substitutions. However, they usually associate identical repeats of the same allele, which can increase the protein production and be selected for (a quantitative advantage). The recent discovery of several heterogeneous duplications implicated in insecticide resistance in mosquitoes provided means to finally test Haldane's hypothesis. Insecticide‐resistant individuals are advantaged in the presence of insecticide (they survive) but endure severe selective costs (their general performances are reduced) compared to susceptible individuals. Using experimental evolution and manipulating the selective pressures (insecticide dose for the advantage and the rearing conditions for the cost), we first showed that balanced antagonist pressures generate overdominance. Introducing a duplicated allele latter showed that it was indeed selected over single copies, as predicted by Haldane. Our study thus shows that heterogeneous duplications associating two already divergent alleles can be selected when the environmental conditions result in trade‐offs favoring overdominance. However, they also create new and already divergent material for genetic innovation and could play a crucial role in the evolution of gene families.

## Introduction

**Figure 1:**
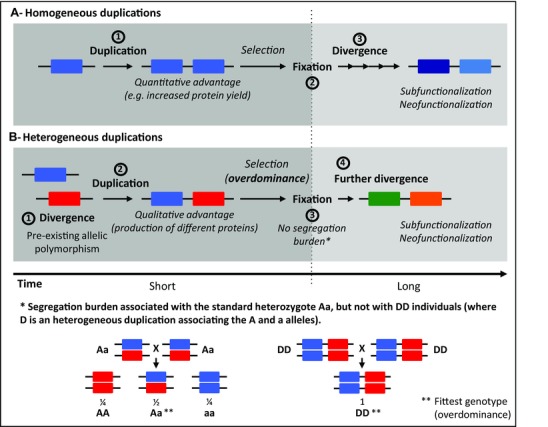
Origins and adaptive fates of the different types of gene duplications. Each rectangle represents one copy of a given locus; the colors indicate that two copies/alleles differ in sequence and are associated with different functions. The timing of the various processes is indicated by the long black arrow, and the background colors differentiate the short and longer term processes. (A) Homogeneous duplications associate two identical copies of a gene (1). They can be selected, for example, for quantitative advantages due to increased protein yield, and get fixed (2). On the long term, they can diverge and acquire new functions (3). (B) In the case of heterogeneous duplications, the allelic divergence (1) precedes the duplication (2). They can be selected in case of overdominance because they allow the production of different proteins, and get fixed because they do not endure the segregation burden associated with standard heterozygotes (the * diagram illustrates the progenies of the two crosses and their proportions) (3). They carry different functions before fixation, but can then further diverge and acquire new functions (4).

Recent genomic studies have shown that gene duplications are widespread, but mostly deleterious (Schrider and Hahn 2010). Some are nevertheless adaptive, and many examples of quantitative advantages (i.e., increased protein production) have been reported associated with identical repeats of a locus (homogeneous duplications) (reviewed in Innan and Kondrashov 2010; Katju and Bergthorsson 2013). Another kind of duplication associates two or more alleles of a single locus (i.e., divergent copies) on the same chromosome (heterogeneous duplications, Fig. 1). Their adaptive role is much harder to assess (Lenormand et al. 1998; Labbé et al. 2007a; Innan and Kondrashov 2010), as only a few examples of recent heterogeneous duplications have been described to date (Labbé et al. 2007a; Djogbénou et al. 2008; Remnant et al. 2013; Sonoda et al. 2014). In 1954, Haldane suggested that they could be selected for by enabling the permanent association of overdominant heterozygous alleles, with no segregation burden (Haldane 1954). In the early 20th century, overdominance (i.e., the situation in which the heterozygote is fitter than either of the homozygotes) was originally put forward as one of several mechanisms underlying the hybrid vigor observed in crosses between inbred strains, and as the only condition allowing a polymorphic equilibrium with constant fitnesses in population genetics equations (Fisher 1922; East 1936; Dobzhansky 1952). Sickle cell anemia is an iconic and much cited example (Lewontin 1974). However, empirical evidence for overdominance in natural populations remains scarce (Hedrick 2012; Llaurens et al. 2017), and its contribution to the selection of heterogeneous duplications has never been assessed.

In several mosquito species, including the malaria vector *Anopheles gambiae* and the West Nile virus vector *Culex pipiens*, the *ace‐1* R resistance allele encodes an acetylcholinesterase protein differing from that encoded by the susceptible S allele by a single amino‐acid substitution (G119S): this substitution prevents organophosphate insecticides (OPs) from binding to their target, resulting in resistance (Weill et al. 2003). However, the G119S substitution also greatly decreases the activity of the protein (Alout et al. 2008), probably accounting for the high selective cost associated with homozygous RR individuals: higher larval mortality, lower fecundity, and lower mating success for RR males than for homozygous SS males (Duron et al. 2006; Assogba et al. 2015, 2016). This situation results in a fitness trade‐off, with the selection of RR individuals in the presence of insecticides, and selection against these individuals in the absence of pesticides. RS individuals have intermediate characteristics, with lower resistance than RR individuals, at a lower cost (Labbé et al. 2014; Assogba et al. 2015). This resistance/cost trade‐off, as well as the migration/selection balance resulting from the alternating treated and nontreated areas, promote *ace‐1* polymorphism (Lenormand et al. 1999; Labbé et al. 2007b).

Gene duplications (D alleles) bringing a susceptible S copy and a resistant R copy together in the same haplotype, on a single chromosome, have been identified in both *A. gambiae* and *C. pipiens* (Lenormand et al. 1998; Labbé et al. 2007a; Djogbénou et al. 2008). In *C. pipiens*, several independent duplications have been identified, and some may fix in natural populations (Labbé et al. 2007a; Alout et al. 2010; Osta et al. 2012). Both R and S copies are identical to single‐copy S and R alleles found in the same populations, so that the D alleles probably result from unequal crossing‐overs in heterozygotes (Labbé et al. 2007a). Most importantly these heterogeneous duplications confer a heterozygote‐like phenotype [RS] (Labbé et al. 2014).

This model thus provides us with a unique opportunity to test Haldane's hypothesis: moderate doses of insecticide could result in balanced selective pressures, where the advantage/cost trade‐off could favor the intermediate performances of heterozygotes, and thus the duplicated alleles.

We used experimental evolution to test these hypotheses. Our study showed that antagonist selective pressures could lead to overdominance, in which case the ultimate allele frequencies depended on the balance between the relative intensities of these selective pressures. It also showed that heterogeneous duplications were indeed selected in these contexts. Half a century later, our study thus provides strong experimental support for a theoretical claim made by one of the fathers of modern evolutionary theory that has long remained untested.

## Methods

### MOSQUITO STRAINS

Three *C. pipiens* mosquito laboratory lines sharing the same genetic background (>99%) were used in this study: Slab (Georghiou et al. 1966), SR (Berticat et al. 2002), and Ducos‐DFix (Labbé et al. 2014). All share the Slab genetic background, but they carry different *ace‐1* alleles: the single‐copy susceptible S (isolated in California), the single‐copy resistance R (isolated in Southern France), and the heterogeneous resistance duplication D (D_1_ allele, isolated in Martinique), respectively. D copies are identical except for the G119S mutation, and differ from the S and R alleles by a few synonymous mutations (Labbé et al. 2007a).

### EXPERIMENTAL EVOLUTION IN POPULATION CAGES

**Figure 2:**
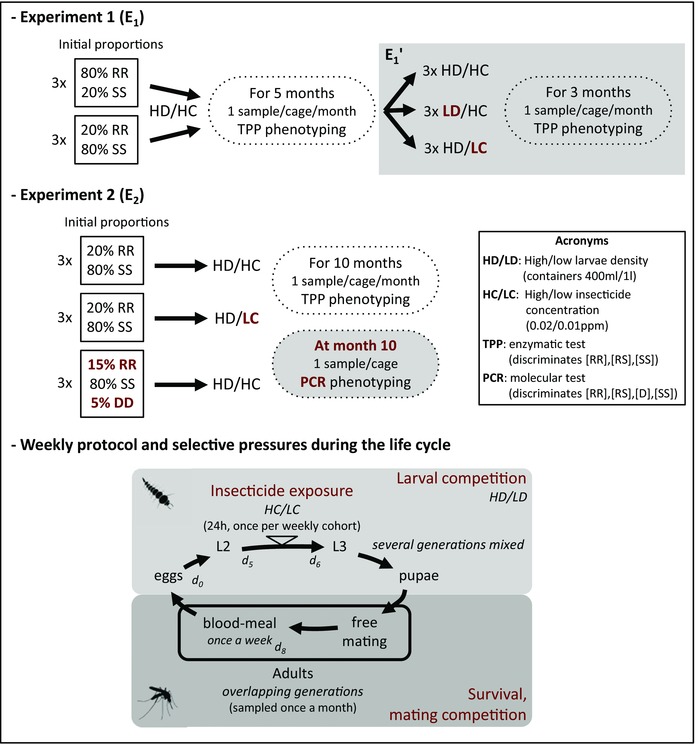
Experimental design. The experimental design of the two experiments (E_1_ and E_2_) is presented (see text also): number of replicates, initial genotype proportions, experimental conditions, duration, sampling design, and phenotyping methods (the acronyms are defined in the box). For experiment E_1_, the replicates were split at month 4 (E_1_′) and the experimental conditions were modified (reference = HD/HC) in the different subsets; the modifications are bolded in red. For experiment E_2_, the conditions differing from the reference (HD/HC) and the alterations to the initial genotype proportions are bolded in red. A specific phenotyping method was used only for the 10th month (bolded in red). The weekly protocol is also represented (see also text); the timing is indicated in days from the egg rafts′ collection (*d_i_*). The various selective pressures are indicated in red and their origins are italicized.

At the start of each experiment replicate, we mixed 500 second‐instar larvae (L_2_) of the different strains in various proportions, to control for any effect of initial conditions or genetic drift. The larvae were reared until the emergence of the adult. The adults were allowed to mate freely and the females were provided with a blood meal (chicken). Thereafter, the experiment was conducted in weekly cycles, under standard conditions (25°C, >60% humidity, 12‐h light:12‐h dark). On the first day (*d_0_*), the egg rafts were collected and placed together in a single container with ∼400 ml of water, resulting in a high larval density (high density or HD, >1000 larvae/l). On day five (*d_5_*), larvae (∼L_2_) were exposed for 24 h to 0.02 ppm of temephos (OP insecticide, Bayer®), the high insecticide concentration (or HC), estimated from bioassays (Labbé et al. 2014). On day six (*d_6_*), the survivors were collected and placed together in a single container in the cage, to allow the adults to emerge; the adults mated freely. On day eight (*d_8_*), the females were provided with a new blood meal. These weekly cycles generated overlapping generations (adults remained in the cage until their death, two to six weeks later) and ensured that all individuals were exposed to the insecticide once in their lives. The control conditions were HD/HC. These conditions were modified by reducing larval density (low density or LD) during rearing, through the use of larger containers (∼1 l), or by reducing the concentration of insecticide (low concentration or LC = 0.01 ppm). The experimental design is summarized in Figure 2. Each month, we sampled an average of 48 adults at random from each cage for the estimation of phenotypic frequencies (total number of individuals analyzed = 6547; Table A1).

### PHENOTYPING

For each sample, individual phenotypes were established with the TPP test (Bourguet et al. 1996) (Table A1), based on the activity of the acetylcholinesterase AChE1 in the presence and absence of insecticide (propoxur, Baygon®). This test discriminates between three phenotypes: [SS], [RR], and [RS]. The first two are always unambiguous and correspond to the homozygous SS and RR genotypes, respectively. When only the R and S alleles are present, the last phenotype corresponds to the standard heterozygote RS (i.e., the phenotypic frequencies correspond to the genotypic frequencies). However, when the D allele is present, the [RS] phenotype becomes ambiguous, as it can result from four genotypes: RS, DD, DS, and DR.

In the last month of the experiment E_2_ (Fig. 2), we distinguished standard heterozygotes (RS) from individuals carrying at least one D allele (DD, DS, and DR; these three genotypes cannot be distinguished), using a PCR test specific for the susceptible copy of the D allele (DucosEx3dir – DucosEx3rev; Labbé *et al*. 2014), directly on second‐instar larvae (no DNA extraction was required, the larvae dissolve in the buffer during the first PCR 95°C denaturation step) (Table A2).

### ESTIMATION OF ALLELE FREQUENCIES

When only the R and S alleles were present, the R allele frequency *f(R)* was calculated directly from the phenotypic frequencies for each generation of each replicate.

However, this was not possible when the D allele was present (see, section "Phenotyping"). In this case, we calculated the apparent R frequency f*(R) = f([RR]) + 0.5f([RS]), that is, as if only R and S were present (D carriers then appear as heterozygotes [RS]).

For the last month of experiment E_2_ (Fig. 2), we inferred the D, R, and S allele frequencies from the phenotypic frequencies (Table A2), assuming panmixia and using the maximum‐likelihood approach developed by Lenormand et al. (1998): for each replicate, we calculated the log‐likelihood *L* of observing the phenotypic data as

$$L\;=\sum_{i}n_{i}ln\left(f_{i}\right)\;,$$

with *n_i_* and *f_i_* the observed number and the predicted frequency of individuals with phenotype *i*, respectively. *L* was then maximized (*L_max_*) with a simulated annealing algorithm (Lenormand et al. 1998). For each allele frequency, the support limits (SL) were then calculated as the minimum and maximum values that this frequency could take without significantly decreasing the likelihood (i.e., *L_max_* −1.96, roughly equivalent to 95% confidence intervals).

### ESTIMATION OF SELECTION COEFFICIENTS

We estimated the relative fitness of the various phenotypes, using a simple deterministic (i.e., no drift) population genetics model considering infinite populations and discrete generations (two per month). This model does not completely reflect our experiments. First, drift did play a role in the allele dynamics, but it had a much smaller impact than selection, which could lead to more dispersion than expected in the observed data; this was handled statistically by controlling for overdispersion in the likelihood model (see below). Second, the generations in the experiment were overlapping, so that the actual generation number is probably below two, which should, conservatively, lead to an underestimation of fitness differences. Hence, our approach was statistically robust to these simplifications.

We used a two‐step model: (1) Reproduction: the frequency *f_gi_* of each genotype *g* among the larvae in generation *i* was calculated from the allele frequencies (*f_R_* and *f_S_* for the R and S alleles, respectively) in the gametes of the previous generation (*i − 1*), assuming panmixia (*f_RRi _ =  f_R(i‐1)_^2^*, *f_SSi _ =  f_S(i‐1)_^2^* and *f_RSi _ =  2f_R(i‐1)_f_S(i‐1)_*); (2) Selection was taken into account between the larval stage and the adult stage, to calculate the frequency *f′_gi_* of each genotype *g* in the adults of generation *i* (*f′_gi_  =  (f_gi_w_g_)/Σ(f_gi_w_g_)*, where *w_g_* is the fitness of the genotype *g*). The fitness of heterozygous individuals, [RS], *wrs* = 1 was used as a reference. The relative fitnesses of the SS and RR genotypes were set as *wss*  =  1 + *sss* and *wrr* = 1 + *srr*, respectively, where *sss* and *srr* are the corresponding selection coefficients. The allelic frequencies in the gametes produced by the surviving adults of generation *i* were then calculated from the genotypic frequencies of these individuals (*f_Ri_  =  f′_RRi _+ 0.5f′_RSi_*, *f_Si_  =  f′_SSi _+ 0.5f′_RSi_*).

This model was adjusted to the data (phenotypic frequencies) through a maximum likelihood approach (R software version 2.15.1 https://www.r-project.org/ package optim, method L‐BFGS‐B). The SL associated with the selection coefficients *sss* and *srr* were calculated from the likelihood profile (*L_max_* −1.96) established from 10^6^ simulations. For each model, we calculated the percentage of the total deviance explained as *%TD* = (*D_max_* − *D_mod_*) / (*D_max_* – *D_min_*) (where *D_min_*, *D_max_*, and *D_mod_*, are the minimal, maximal, and model deviances, respectively, with *D* = −2 *L*), and the overdispersion as *od*  =  *D_res_* / *df_res_* (where *D_res_* and *df_res_* are the residual deviance and the residual degrees of freedom, respectively). We assessed the significance of differences in selection coefficients between rearing conditions (HC/HD, HC/LD, and LC/HD), by adjusting a complete model with two parameters for each set of conditions considered (*sss* and *srr*). A simplified model, with only one *sss* or *srr* parameter for all conditions, was then computed. The two models were then compared using likelihood ratio tests corrected for overdispersion (*LRT_od_*): when significant, the simplified model was rejected, and the tested selection parameter was considered to differ significantly between the conditions tested.

## Results and Discussion

### OVERDOMINANCE RESULTS FROM BALANCED ANTAGONIST SELECTIVE PRESSURES

We first investigated the possibility that intermediate insecticide doses could result in overdominance by setting up six population evolution experiments in cages containing a mixture of *C. pipiens* RR and SS genotypes, with three replicates having an initial R frequency *f_0_(R) * =  0.8 and three replicates having an initial *f_0_(R)* = 0.2 (experiment E_1_, Fig. 2). Each new generation was exposed to a high concentration (HC) of insecticide that killed almost all SS individuals, a few RS but no RR individuals, thereby favoring selection of the R allele. The larvae were also reared at HD, to increase competition, resulting in higher levels of larval mortality. The emerging adults were released into the same cage: they mated freely and were able to reproduce every week until their death (overlapping generations), favoring the selection of individuals with longer lifespans and higher mating success, that is, those carrying the less costly S allele. We monitored changes in R allele frequency *f(R)* every month (about two overlapping generations), by genotyping adults randomly sampled from each of the six cages (there are only RR, SS, and RS individuals; Fig. 2 and Table A1). If these conditions result in overdominance, it is possible to predict the dynamics of the alleles: (1) *f(R)* should reach a stable equilibrium ($\hat{f}$ *(R)*): even when RS is the fittest genotype, when two heterozygotes mate, only half of their progeny carries the RS genotype (i.e., there is a segregation burden), the other half carrying the less fit genotypes RR or SS; the polymorphism is thus stable; (2) the frequency at equilibrium should depend on the relative fitness of each genotype in the environmental conditions considered, but should be independent of the initial frequencies of the alleles (*f_0_*).

**Figure 3:**
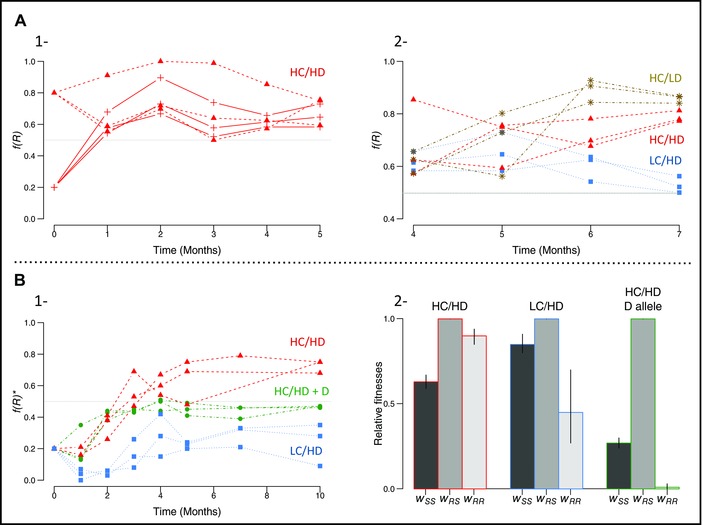
**R allele dynamics in the various experimental evolution studies and fitness estimations** **(A)** Changes in R allele frequency *f(R)* in E_1_ (Table 1). (A**1)** Original rearing conditions at high density (HD) and high insecticide concentration (HC), R and S alleles only, with *f_0_(R)* = 0.8 (triangles, dashed lines) or *f_0_(R)* = 0.2 (plus signs, solid lines). (A**2)** Altered rearing conditions after four months (E_1_’, Table 1): controls (HD/HC; red triangles, dashed lines), reduced density (LD/HC; brown stars, dotted‐dashed lines), reduced insecticide concentration (HD/LC; blue squares, dotted lines). **(B1)** Changes in *f(R)* in E_2_ (Table 1): controls (HD/HC, R and S; red triangles, dashed lines), reduced insecticide concentration (HD/LC, R and S; blue squares, dotted lines), duplicated allele assay, that is, control rearing conditions (HD/HC), R, S, and D alleles, with *f_0_(D)* = 0.05, *f_0_(R)* = 0.15, and *f_0_(S)* = 0.8 (*NB: in this set up, as all genotypes carrying D confer a heterozygote phenotype, *f(R)* is estimated as if only R and S were present, see text). (b**2)** The relative fitnesses of the [SS] (*w_SS_*, dark gray bars), [RS] (*w_RS_* = 1, medium gray bars), and [RR] (*w_RR_*, light gray bars) phenotypes (see “Estimation of selection coefficients” in section Methods) are presented for the different conditions of the E_2_ experiment (B1 plot colors are conserved).

This is what we observed, despite variations due to drift and sampling: the frequency of R in all replicates ultimately converged around $\hat{f}$ *(R)* = 0.68 ± 0.08 (mean ± standard deviation), regardless of its initial frequency (Fig. 3A1). Estimates based on a population genetics model confirmed that the relative fitness of the heterozygotes (*w_RS_*) was significantly higher than those of the two homozygotes (confirming overdominance) and that selection for heterozygotes was strong (E_1_, Table 1): RR individuals were fitter than SS individuals (as expected from $\hat{f}$ *(R)* > 0.5), but this resistance advantage did not compensate for the fitness cost relative to RS mosquitoes.

**Table 1:** Experimental design and relative fitness estimations

	Allele	Conditions	*W_SS_ (SL)*	*W_RR_ (SL)*	*%TD*	*od*
**E_1_**	*S* / *R*	*HC*/*HD*	***0.20 (0.16–0.24)***	***0.58 (0.54–0.62)***	*0.66*	*5.55*
**E_1_’**	*S* / *R*	*HC*/*HD*	***0.10 (0.04–0.21)***	***0.69 (0.62–0.76)***	*0.85*	*2.8*
	S / R	HC/**LD**	**0.59 (0.35*–*0.90)**	**1.22 (1.09*–*1.39)**	0.89	2.73
	S / R	**LC**/HD	0.91 (0.68***–***1.04)	**0.78 (0.67*–*0.89)**	0.84	2.16
**E_2_**	*S* /*R*	*HC*/*HD*	***0.63 (0.59–0.67)***	***0.90 (0.85–0.94)***	*0.76*	*3.04*
	S / R	**LC**/HD	**0.85 (0.80*–*0.91)**	**0.45 (0.27*–*0.70)**	0.73	5.41
	S / R / **D**	HC/HD	**0.27 (0.24*–*0.30)**	**0.01 (0.00*–*0.03)**	0.89	3.54

For the various evolution experiments (E_i_), the alleles in competition are indicated (single‐copy susceptible S, single‐copy resistant R, and heterogeneous duplication D). The conditions in which the larvae were reared are also indicated: high or low insecticide concentration (HC = 0.02 ppm and LC = 0.01 ppm temephos, respectively) and high or low larval density (HD or LD, respectively); controls are indicated in italics; conditions differing from the controls are shown in bold. For each set of conditions, a population genetics model was used to calculate the fitnesses of the single‐copy susceptible (*wss*) and resistance (*wrr*) homozygotes relative to that of the heterozygote (*wrs* = 1). When the associated support limits (SL, in brackets) include 1, *w_SS_* and/or *w_RR_* are not significantly different from *w_RS_*; significant differences are shown in bold. The percentage of the total deviance explained by each model (*%TD*) and its overdispersion (*od*) are also indicated.

We then confirmed that the frequencies at equilibrium were constrained by the evolutionary trade‐offs of the various genotypes by altering the environmental conditions. After four months, we kept three of the cages in the original conditions (controls: HD, HC), but the other three cages were each split in two (experiments E_1_′, Table 1, Fig. 2). In one half of these three cages, we reduced larval rearing density (low density, LD), but maintained the HC of insecticide treatment (Fig. 2). These conditions were expected to reduce the competition between larvae, and thus, the cost of the R allele. As expected, *f(R)* increased (Fig. 3A2, brown stars). Accordingly, the relative fitness ranking changed significantly (LRT_od_, *F* = 10.1; *Δdf* = 2; *P* < 0.001): RR became the fittest genotype (E_1_’, Table 1), but RS remained fitter than SS. In the other half of these three cages, we reduced the insecticide concentration (LC), but maintained the high larval density (HD) (Fig. 2). These conditions were expected to reduce the selective advantage of the R allele. As expected, *f(R)* decreased (Fig. 3A2, blue squares). Due to the shorter duration, equilibriums were not reached: the selective coefficient estimates obtained are less precise, RS remained the fittest genotype, but SS became slightly fitter than RR (LRT_od_, *F* = 32.7; *Δdf* = 2; *P* < 0.001; E_1_’, Table 1). These experiments thus provided evidence that environmental modifications affecting the selective advantage or the cost can alter the frequency equilibrium.

We assessed the robustness of these conclusions by replicating the study described above. Three cages were set up as controls (HD, HC), with an initial *f_0_(R)* = 0.2 (experiment E_2_, Fig. 2): after 10 months, the mean R frequency had reached an equilibrium at $\hat{f}$ *(R)* = 0.73 ± 0.04 (Fig. 3B1, red triangles), similar to that in E_1_ ($\hat{f}$ *(R)* = 0.68 ± 0.08; Student's *t* test, *P* = 0.34). This robustness (nine cages stabilizing at about the same frequency, regardless of their initial frequency) confirmed that the allele dynamics in our experiments were mostly driven by selection. In parallel, three other cages were set up with a low insecticide concentration (HD, LC), again with an initial *f_0_(R)* = 0.2 (E_2_, Fig. 2). The mean R frequency had also reached equilibrium by 10 months, but at a lower value, $\hat{f}$ *(R)* = 0.24 ± 0.14 (Fig. 3B1, blue squares). As expected, model estimates confirmed that RS individuals were the fittest in both conditions (Table 1). However, fitness ranking changed according to the different conditions (LRT_od_, *F* = 172.7; *Δdf* = 2; *P* < 0.001; Table 1): in control (HD, HC) conditions, RR individuals were fitter than SS individuals (*wrs>wrr>wss*), whereas SS individuals were fitter than RR individuals in LC conditions (*wrs>wss>wrr*), indicating that RR resistance relative advantage cannot compensate its cost at lower insecticide concentrations. E_2_ thus confirmed the conclusions of E_1_: different overdominance equilibria may exist, depending on selective pressure intensities, which can alter the relative fitness trade‐offs of the different genotypes (providing a clear alternative example of overdominance to sickle cell anemia; Lewontin 1974).

### OVERDOMINANCE FAVORS THE HETEROGENEOUS DUPLICATED ALLELE

We then tested whether the heterogeneous *ace‐1* duplication (D) was favored when the environmental conditions result in overdominance. Previous studies suggested that D alleles could confer fitness trade‐offs on their carriers similar to those of standard RS heterozygotes (Labbé et al. 2014; Assogba et al. 2015). Moreover, DD individuals should not suffer from the segregation burden associated with the RS genotype. We set up three replicates in control conditions (HC, HD), but introduced a D allele, such that *f_0_(D)*  =  0.05, *f_0_(R)*  =  0.15, *f_0_(S)*  =  0.80 (E_2_, Fig. 2). If D is indeed favored, then most individuals should be phenotypic heterozygotes [RS], with an apparent R frequency at equilibrium $\hat{f}$ (R)* close to 0.5 (see, section Methods).

This is precisely what we observed: after 10 months, most of the individuals were indeed [RS] and no [RR] individuals were observed ($\hat{f}$ *(RS)*  =  0.93 ± 0.02), with $\hat{f}$ (R)* = 0.47 ± 0.01 (Fig. 3B1, green circles). The estimated relative fitnesses of [SS] and [RR] were both close to 0 (E_2_, Table 1 and Fig. 3B2). The persistence of a few [SS] individuals while all [RR] disappeared suggests a slightly asymmetrical trade‐off: as phenotyping preceded selection, it could result from a higher fitness of DS than DR individuals, thereby still generating a few new [SS] individuals. D invasion was further confirmed with a specific molecular test applied to about 90 individuals from each replicate: the frequency of this allele increased from 0.05 to an estimated $\hat{f}$ *(D)* = 0.72 ± 0.07 (Table A2). All the [RS] individuals carried D (i.e., no standard RS heterozygotes were found in the cages); the [RS] phenotype fitness therefore corresponded to genotypes DS, DR, or DD. Heterogeneous duplications, by conferring the heterozygous phenotype without the associated segregation burden, can thus be fixed when selective trade‐off favors overdominance, that is, when antagonist selective pressures are balanced.

### OVERDOMINANCE IS PROBABLY COMMON BUT TRANSITORY

Overdominance is almost certainly more widespread than generally thought (Hedrick 2012; Llaurens et al. 2017): (i) multivariate stabilizing selection probably frequently leads to overdominance for new mutations that are beneficial in the heterozygous state (Manna et al. 2011), and (ii) recent adaptation usually involves trade‐offs (Orr 2005), so balanced antagonist selective pressures would result in overdominance; some studies measuring the fitness associated to new mutations in the laboratory have confirmed these expectations (Peters et al. 2003). Our study contributes to explain the discrepancy between the prevalence of overdominance in newly arising mutations and the relative rarity of segregating overdominant alleles in the field (Manna et al. 2011): it shows that overdominance may not be robust, as limited modifications of the environment can favor one allele over the other; more importantly, we showed that, as predicted by Haldane (1954), overdominance can be rapidly abolished by the occurrence and selective spread of a heterogeneous duplication, a situation that has probably contributed to the scarcity of persistent cases of overdominance in natural populations.

### HETEROGENEOUS DUPLICATIONS CAN BE IMMEDIATELY ADAPTIVE AND COULD FUEL FUTURE EVOLUTION

Overdominance (and more generally heterozygote advantage) has been proposed to explain the diversification of several multigenic families, such as MHC genes in vertebrates (Spurgin and Richardson 2010), R genes in plants (Michelmore and Meyers 1998; Panchy et al. 2016), and MAT genes in basidiomycetes (May et al. 1999): heterozygotes display a higher fitness because they can resist to more pathogens (MHC and R genes) or mate with more sexual types (MAT). However, these duplications are ancient and it is difficult to determine whether copy sequence polymorphism existed before the duplications (i.e., heterogeneous duplications associating existing alleles) or resulted from postduplication divergence (i.e., originally homogeneous duplications; Fig. 1). The difficulty to identify ancient heterogeneous duplications probably explains why their potentially crucial adaptive role has been overlooked.

Fortunately, a handful of contemporaneous heterogeneous duplications have been described that allow assessing how and why they are selected. Interestingly, they all concern insecticide target genes, probably because these duplications are recent (occurring in response to a few decades of anthropic environmental modification), associated with irreducible trade‐offs, and highly scrutinized due to their impact on vector control and public health (Labbé et al. 2007a; Djogbénou et al. 2008; Remnant et al. 2013; Sonoda et al. 2014). The *ace‐1* duplications in *C. pipiens* remain however the most deeply studied: so far, at least 13 duplicated alleles have been identified, most of them resulting from independent duplication events (Labbé et al. 2007a; Alout et al. 2010; Osta et al. 2012). This suggests that the conditions for overdominance are probably quite frequent: insecticide treatment practices typically result in a patchy environment, with alternating treated and nontreated areas: if the grain of the environment is smaller than the dispersal distance of the mosquito, it could result in marginal overdominance (Lenormand et al. 1998; Labbé et al. 2007b), or even full overdominance if field conditions result in low insecticide doses (this study). Moreover, the treatment intensities typically vary in time, as they are applied usually only in some periods of the year (Lenormand et al. 1999): these fluctuations of antagonist selective pressures could also on average favor the heterozygote phenotype (i.e., marginal overdominance resulting from fluctuating selection). This frequent selection of D alleles could seriously hinder mosquito control: because they display a lower cost than R, these resistance alleles could make OP and CX insecticides virtually obsolete and threaten control strategies based on insecticide alternation. This is particularly pressing in the case of *A. gambiae*, the malaria vector, where these insecticides have been suggested as replacements for the widely used pyrethroids that face high and widespread resistance (Assogba et al. 2015, 2016).

This unique example shows that heterogeneous duplications can result in an immediate qualitative advantage in fluctuating or patchy environments, or more generally in environments with balanced antagonistic selective pressures. As predicted by Haldane in 1954, we indeed demonstrated that these duplications can be selected because they allow the permanent association of overdominant heterozygous alleles, with no segregation burden (Haldane 1954). These properties could prove useful for breeders (plant breeders in particular), as new genome‐editing tools (e.g., CRISPR‐cas9; Sander and Joung 2014) could be used to generate heterogeneous duplications to create stable lines displaying specific heterosis otherwise found only in hybrids (Fu et al. 2014).

However, heterogeneous duplications should also be studied in more detail in terms of their role in long‐term evolution, as they probably bear witness to ancestral situations of transitory overdominance. As homogeneous duplications, they create new material for genetic innovation (Lynch and Force 2000; Osada and Innan 2008; Innan and Kondrashov 2010). However, as they result from the association of two already divergent alleles, their dissimilar copies immediately carry different functions, that is, copy functional divergence precedes fixation (Fig. 1). They are thus more likely to evolve further through subfunctionalization and neofunctionalization, and should do it more rapidly than homogeneous duplications; these can be first selected, for example, for increased protein quantity, but would diverge later (Lenormand et al. 1998; Labbé et al. 2007a; Innan and Kondrashov 2010). These heterogeneous duplications could thus play a major, albeit yet underestimated, role in the evolution of gene families.

Associate Editor:

Handling Editor:

## Appendix

**Table A1:** Population cages phenotypic data

			Replicate 1				Replicate 2				Replicate 3			
	Alleles and conditions	Month	[SS]	[RS]	[RR]	Tot	[SS]	[RS]	[RR]	Tot	[SS]	[RS]	[RR]	Tot
E_1_	S / R HC / HD	1	0	41	4	45	5	17	20	42	4	30	11	45
		3	1	24	23	48	0	10	38	48	1	30	17	48
		3	5	28	12	45	1	21	22	44	6	31	8	45
		4	5	27	16	48	2	29	17	48	3	34	11	48
		5	6	22	20	48	0	26	22	48	4	32	12	48
			Replicate 4				Replicate 5				Replicate 6			
		Month	[SS]	[RS]	[RR]	Tot	[SS]	[RS]	[RR]	Tot	[SS]	[RS]	[RR]	Tot
		1	1	38	6	45	3	31	11	45	0	8	37	45
		3	4	21	23	48	3	21	24	48	0	0	48	48
		3	3	19	3	25	3	25	15	43	0	1	43	44
		4	9	23	16	48	0	36	12	48	1	12	35	48
		5	1	19	23	43	0	39	9	48	2	12	18	32

			Replicate 1				Replicate 2				Replicate 3			
	Alleles and conditions	Month	[SS]	[RS]	[RR]	Tot	[SS]	[RS]	[RR]	Tot	[SS]	[RS]	[RR]	Tot
E_1_′	S / R HC / HD	4	9	23	16	48	1	12	35	48	0	36	12	48
		5	1	19	23	43	2	12	18	32	0	39	9	48
		6	0	21	27	48	1	29	18	48	1	27	20	48
		7	0	18	30	48	0	5	6	11	0	19	24	43
	S / R HC / LD	4	9	23	16	48	2	29	17	48	0	36	12	48
		5	5	16	27	48	0	19	29	48	8	26	14	48
		6	1	13	34	48	0	9	39	48	0	7	41	48
		7	0	15	32	47	0	13	35	48	0	12	33	45
	S / R LC / HD	4	5	27	16	48	2	29	17	48	3	34	11	48
		5	6	22	20	48	0	26	22	48	4	32	12	48
		6	4	14	6	24	8	19	21	48	6	24	18	48
		7	13	22	13	48	8	28	10	46	8	26	14	48

			Replicate 1				Replicate 2				Replicate 3			
	Alleles and conditions	Month	[SS]	[RS]	[RR]	Tot	[SS]	[RS]	[RR]	Tot	[SS]	[RS]	[RR]	Tot
E_2_	S / R HC / HD	1	39	19	0	58	39	19	0	58	28	20	0	48
		2	12	36	0	48	26	19	3	48	11	35	2	48
		3	6	33	9	48	11	29	8	48	3	24	21	48
		4	1	36	11	48	1	26	16	43	4	36	8	48
		5	3	24	21	48	1	22	25	48	5	11	4	20
		6	–	–	–	–	–	‐	–	–	–	–	–	–
		7	–	–	–	–	0	14	20	34	–	–	–	–
		8	–	–	–	–	–	–	–	–	–	–	–	–
		9	–	–	–	–	–	–	–	–	–	–	–	–
		10	2	57	37	96	2	44	50	96	0	48	48	96
	S / R HC / LD	1	48	2	1	51	47	0	0	47	41	7	0	48
		2	42	6	0	48	42	6	0	48	45	3	0	48
		3	27	17	4	48	40	8	0	48	35	12	1	48
		4	16	24	8	48	21	27	0	48	34	14	0	48
		5	25	23	0	48	15	13	0	28	28	19	0	47
		6	–	–	–	–	–	–	–	–	–	–	–	–
		7	9	14	1	24	17	31	0	48	27	19	0	46
		8	–	–	–	–	–	–	–	–	–	–	–	–
		9	–	–	–	–	–	–	–	–	–	–	–	–
		10	30	64	2	96	42	52	0	96	79	17	0	96
	S / R / D* HD / HC	1	35	13	0	48	15	34	0	49	36	12	0	48
		2	12	36	0	48	7	41	0	48	5	26	1	32
		3	5	43	0	48	7	41	0	48	6	42	0	48
		4	3	23	0	26	0	57	1	58	1	46	1	48
		5	6	40	1	47	1	47	0	48	9	39	0	48
		6	–	–	–	–	–	–	–	–	–	–	–	–
		7	–	–	–	—‐	4	44	0	48	11	37	0	48
		8	–	–	–	–	–	–	–	–	–	–	–	–
		9	–	–	–	–	–	–	–	–	–	–	–	–
		10	9	130	0	139	12	128	0	139	7	129	0	136

The number of individuals for each phenotype ([RR], [RS], [SS], TPP test) is indicated for each replicate and each month. The total number of individuals tested (Tot) was usually 48, but varied due to technical issues.

*Due to the presence of the D allele, the [RS] phenotype corresponds to RS, DS, DR, or DD genotypes in these replicates.

**Table A2:** Phenotypic data used to estimate D frequency at month 10 in E_2_

Phenotype*	Replicate 1	Replicate 2	Replicate 3
[RR]	0	0	0
[RS]	0	0	0
[D]	83	80	86
[SS]	7	11	4
Tot	90	91	90

The number of individuals for each phenotype ([RR], [RS], [D], [SS]) and the total number of tested individuals (Tot) are indicated for each replicate.

*All phenotypes correspond to only one genotype, except [D] that corresponds to DS, DR, or DD genotypes.
